# A Causality-guided Statistical Approach for Modeling Extreme Mei-yu Rainfall Based on Known Large-scale Modes—A Pilot Study

**DOI:** 10.1007/s00376-022-1348-3

**Published:** 2022-05-14

**Authors:** Kelvin S. Ng, Gregor C. Leckebusch, Kevin I. Hodges

**Affiliations:** 1 School of Geography, Earth and Environmental Sciences, University of Birmingham, Birmingham, B15 2TT UK; 2 Department of Meteorology and NCAS, University of Reading, Reading, RG6 6BB UK

**Keywords:** extreme rainfall, Mei-yu front, causality-guided approach, large-scale climate modes, 极端降雨, 梅雨锋, 因果关系引导方法, 大尺度气候模式

## Abstract

Extreme Mei-yu rainfall (MYR) can cause catastrophic impacts to the economic development and societal welfare in China. While significant improvements have been made in climate models, they often struggle to simulate local-to-regional extreme rainfall (e.g., MYR). Yet, large-scale climate modes (LSCMs) are relatively well represented in climate models. Since there exists a close relationship between MYR and various LSCMs, it might be possible to develop causality-guided statistical models for MYR prediction based on LSCMs. These statistical models could then be applied to climate model simulations to improve the representation of MYR in climate models.

In this pilot study, it is demonstrated that skillful causality-guided statistical models for MYR can be constructed based on known LSCMs. The relevancy of the selected predictors for statistical models are found to be consistent with the literature. The importance of temporal resolution in constructing statistical models for MYR is also shown and is in good agreement with the literature. The results demonstrate the reliability of the causality-guided approach in studying complex circulation systems such as the East Asian summer monsoon (EASM). Some limitations and possible improvements of the current approach are discussed. The application of the causality-guided approach opens up a new possibility to uncover the complex interactions in the EASM in future studies.

**Electronic supplementary material:**

Supplementary material is available in the online version of this article at 10.1007/s00376-022-1348-3.

## 摘 要

极端梅雨降雨 (MYR) 会对中国的社会和经济发展造成灾难性影响. 尽管气候模型在模拟气候上已有重要进展, 但这些模型通常难以模拟局部到区域的极端降雨过程(例如, 极端梅雨降雨). 目前来说, 气候模型能较好地模拟大尺度气候模式(LSCM). 由于 极端梅雨降雨 与不同的 大尺度气候模式 之间存在密切关系, 因此, 我们有可能基于大尺度气候模式来构建因果关系引导的数据模型. 该数据模型有望应用于气候模型的模拟以改善其在模拟极端梅雨降雨中的表现. 在此探索性研究中, 我们证明了基于因果关系引导方法的数据模型可以通过已知的大尺度气候模式来建立, 并有效模拟极端梅雨降雨. 该统计模型內的预测变量的相关性以及时间分辨率的重要性都与文献一致. 该研究结果证实了因果关系引导方法在研究东亚夏季风 (EASM) 等复杂环流系统中的可靠性. 本文也讨论了当前方法的一些局限性和可能的改进方法. 因果关系引导方法的应用为未来研究复杂的东亚夏季风中的相互作用开辟了新的可能性.

**Article Highlights:**

- Skillful spatiotemporal statistical models of extreme Mei-yu rainfall can be produced using the causality approach.
- Based on spatial consistency, the large-scale climate modes that are relevant to the regional extreme Mei-yu rainfall can be identified.

## 1. Introduction

Meteorological extreme events affect economic development and societal welfare in an extraordinary way. Many countries in East Asia are affected by a variety of natural meteorological related hazards, e.g., tropical cyclones and extreme rainfall events associated with the Mei-yu front (MYF) embedded in the East Asian Summer Monsoon (EASM) system. It is estimated by the Chinese government that the direct economic losses caused by extreme meteorological events is about 1%–3% of gross domestic product (GDP) every year (Sall, 2013). The exposure could further increase due to the rapid economic development and migration patterns in China. In 2020, record-breaking amounts of extreme Mei-yu rainfall (MYR) were observed over the Yangtze River Valley region (YV) (Liu et al., 2020). This led to severe flooding in the YV, more than 140 casualties, and more than 82 billion renminbi (RMB) direct economic loss (Gan, 2020). Given the enormous impact of MYR, it is necessary to deepen our understanding of various aspects of the MYR, including the onset, duration, and intensity of rainfall (Ding et al., 2021). This could then increase our capability to predict MYR for the near- (e.g. Martin et al., 2020) and the long-term future. Consequently, this will allow policy and decision makers to be able to develop optimal disaster risk reduction and mitigation strategies to strengthen the resilience of society and minimize the impact of meteorological extremes on society.

The MYF is one of the main features of the EASM. The MYF rain band moves in a stepwise manner from low to higher latitudes as the EASM advances northwards from May to August (Ding and Chan, 2005; Wang et al., 2009; Li et al., 2018). While significant improvements have been made in forecasting Mei-yu rainfall using dynamical models on seasonal time scales (e.g. Martin et al., 2020), the accurate prediction of MYR in climate models remains one of the major challenges in the scientific community. This is partially due to coarse climate model spatial resolution, whereby key processes on relevant spatial and temporal scales are not well represented.

Many studies in the past have found a significant association between MYR and several large-scale climate modes (LSCMs), including the Indian Summer Monsoon (ISM) (Wang and LinHo, 2002; Liu and Ding, 2008), the western North Pacific subtropical high (WNPSH) (Zhou and Wang, 2006; Ninomiya and Shibagaki, 2007; Liu and Ding, 2008; Sampe and Xie, 2010; Ding et al., 2021), the South Asian High (SAH) (Liu and Ding, 2008; Ning et al., 2017), the El Nino–Southern Oscillation (ENSO) (Wu et al., 2003; Wang et al., 2009; Ye and Lu, 2011), the Pacific–Japan teleconnection pattern (PJ) (Kosaka et al., 2011; Ding et al., 2021), sea surface temperature anomaly (SSTA) in the Indian Ocean and the South China Sea (e.g. Zhou and Wang, 2006), as well as middle-to-high-latitude features (e.g., the Silk Road Pattern; SRP) (Wang and He, 2015; Ning et al., 2017). Based on these associations, different theories and hypotheses for the underlying mechanisms that control different aspects of MYR have been proposed (e.g., Ding and Liu, 2008; Sampe and Xie, 2010; Ning et al., 2017; Li et al., 2018). Given that many LSCMs have been found to be important in controlling the MYF and MYR, one idea is to construct statistical prediction models for MYR based on relevant LSCMs, because LSCMs are better simulated by climate models (Flato et al., 2013). However, due to the complexity of the EASM system and the limitations of traditional approaches, e.g., the correlation-based approach, in processing large amounts of information, it is difficult to reliably identify robust and comprehensive relationships between MYR and LSCMs. Consequently, this remains as a major challenge.

In recent years, data-driven, causality-guided approaches have started to gain attention from the climate community (Ebert-Uphoff and Deng, 2012; Runge et al., 2012a, b, 2014, 2019a, b; Chaudhary et al., 2016; Kretschmer et al., 2016, 2017; Di Capua et al., 2019). Kretschmer et al. (2017) and Di Capua et al. (2019) demonstrated the advantages of using a causality-guided approach (response-guided causal precursor detection; RG-CPD), to construct statistical forecast models for meteorological extremes. RG-CPD is a two-step procedure. The first step is to construct relevant climate indices, which are related to the response, from the data field using the response-guided community detection algorithm (Bello et al., 2015). This is done by first calculating lagged correlation maps between climate variables and the response. Then the adjacent grid points with significant correlations of the same sign with the response form so-called precursor regions. Climate indices are then constructed by area-weighted averages over the precursor regions. The second step is to apply a causal discovery algorithm (Spirtes et al., 2001; Runge et al., 2012a, 2014, 2015; Runge, 2015) to explore the causal relationship between the climate indices, which are found in the first step. The statistical models constructed, using RG-CPD, by Kretschmer et al. (2017) and Di Capua et al. (2019) have been shown to have excellent performance in predicting extreme stratospheric polar vortex states and Indian summer monsoon rainfall, respectively. This shows the potential of the causality approach in applying it to the prediction on half-monthly to seasonal time scales of other meteorological and climatological extremes as well as exploring underlying physical processes. Our work aims to explore the applicability of the causality approach for predicting MYR based on known LSCMs.

Traditionally, predictors in statistical models are chosen based on the association with the responses, i.e., correlation. However, correlation-based statistical models may have no physical meaning because correlation does not imply causation. In addition, all predictors, which describe different stages of the same process, would be chosen if the traditional association criteria are used. Consequently, correlation-based statistical models suffer from overfitting due to non-causal relationships between predictors and response (Kretschmer et al., 2017). This also implies that these models cannot be applied to alternative data as the models are built using specific datasets. On the other hand, a causality-guided statistical model (CGSM) does not have these drawbacks because it captures the underlying physical relations.

In this pilot study, we aim to answer the following questions: (1) How well do data-driven CGSMs, constructed using known indices of LSCMs, perform in predicting MYR both spatially and temporally? (2) What are the limitations of data-driven CGSMs? In this paper, we demonstrate the usefulness of a data-driven, causality-guided approach, which allows us to explore the relationship between MYR and LSCMs in a more comprehensive and efficient manner. The models constructed based on the predictors, which are selected using this approach, will be shown to have remarkable explanatory power. Due to the property of causality-guided models as discussed above, i.e., CGSMs do not suffer from overfitting due to non-causal relationships between predictors and response, the prediction model can be applied to different datasets for the same phenomena. This opens up the possibility to apply this type of statistical model to climate data that are generated from different models and simulations. Consequently, useful and timely information would be available to policy and decision makers. The paper is arranged as follows: the datasets that are used in this study are outlined in section 2. The methodology is described in section 3. In section 4, we present the main results, including the performance of the models. The discussion about the current approach as well as its limitations can be found in section

5. Conclusions are presented in section 6.

## 2. Data

For model development, the fifth generation European Centre for Medium-Range Weather Forecasts (ECMWF) reanalysis data (ERA5) (Hersbach et al., 2020) is used. ERA5 is produced based on the Integrated Forecasting System (IFS) Cy41r1 with 4D-Var data assimilation. The model resolution is T639 (~32 km) with 137 vertical levels. In this study, ERA5 data from 1979–2018 with spatial resolution of 0.25° × 0.25° is used. Historical rainfall observation data (1961–2018) is obtained from the high resolution (0.25° × 0.25°) gridded observed daily precipitation data from the China Meteorological Administration known as the CN05.1 data set (Wu and Gao, 2013). Similar to the development of the earlier gridded observation data (Xu et al., 2009), the CN05.1 dataset was constructed by interpolation of data from more than 2400 observation stations in China using the "anomaly approach" (Wu and Gao, 2013). In the anomaly approach, a gridded climatology is first calculated, and then a gridded daily anomaly is added to the climatology to obtain the final dataset. The CN05.1 data in the period of 1979–2018 are used for model construction. To examine the limitation of this approach for different climate states, CN05.1 and ERA5 back extension (BE) (preliminary version) (Bell et al., 2020a, b) in the period of 1961–78 are also used.

## 3. Method

### 3.1. *Indices of LSCM*

Indices of LSCMs are calculated using ERA5 (Hersbach et al., 2020) and ERA5 BE (preliminary version) (Bell et al., 2020a, b) for the period 1979–2018 and 1961–78, respectively. Table 1 shows the list of indices of LSCMs considered in this study. Since there are some reported biases in ERA5 BE (ECMWF, 2021), consistency checks between the indices of LSCM calculated from ERA5 and ERA5 BE have been performed. In general, indices of LSCM calculated using ERA5 BE are climatologically consistent with ERA5, except for the area index of the South Asian High (SAHI-Area). The mean and standard deviation of SAHI-Area in ERA5 BE for the period 1961–78, which are approximately equivalent to $1.10 \times 10^7$ km$^2$ and $5.72 \times 10^6$ km$^2$, respectively, are climatologically smaller than those in ERA5, which are approximately equivalent to $1.54 \times 10^7$ km$^2$ and $7.70 \times 10^6$ km$^2$, respectively. This is related to the decadal shift of SAH intensity around the late 1970s (e.g. Xue et al., 2015).

### 3.2. *MYF detection and MYR identification*

The MYF detection scheme was developed in the FOREX project (http://www.birmingham.ac.uk/research/activity/environmental-health/projects/forex.aspx; Access date: 13 December 2021) and was first presented in Befort et al. (2016), Leckebusch et al. (2016), and Befort et al. (2017). This detection scheme is an extension of the Baiu front detection scheme, which was developed by Tomita et al. (2011), to identify the Mei-yu-Baiu front over a large region from China to Japan. The main difference in the detection schemes is that Tomita et al. (2011) used the minimum of the meridional gradient of daily equivalent potential temperature at 850 hPa ($d\theta_e$) to locate the position of the MYF, whereas the minimum of the product of $d\theta_e$ and specific humidity at 850 hPa ($qd\theta_e$) was used by Befort et al. (2016) and Befort et al. (2017). The reason for the difference is that the position of the MYF that is detected using the minimum $d\theta_e$ appears to be too far north in comparison to the rainfall over eastern China (Befort et al., 2016, 2017). Tomita et al. (2011) pointed out that the Mei-yu rainfall over eastern China depends on instability, which is linked to $d\theta_e$, and total amount of humidity. In order to include the effect of moisture, $qd\theta_e$ is chosen here, as in Befort et al. (2016) and Befort et al. (2017), for MYF detection.

Figure 1 shows a flowchart for the MYF detection scheme, and a brief description of this scheme is stated as follows: (1) The 15-day running mean of daily equivalent potential temperature at 850 hPa ($\theta_e$) is calculated to remove sub-synoptic-scale disturbances. (2) $qd\theta_e$ is then calculated on a T63 grid (192 × 96, ~200 km), and values with $\theta_e < 310$ K are masked. (3) An MYF exists if there exist coherent clusters of $qd\theta_e$ minima exceeding 10 grid points that are in a specific region, as stated in Fig. 1 and shown in Fig. S1 in the electronic supplementary material (ESM). (4) The position of the MYF is delineated by a cubic smoothing spline fitted to the grid point values identified from the previous step. Figure 2 shows the climatological positions of the MYF in ERA5. The northward propagation of the MYF from May to August, as described in the literature (e.g., Ding and Chan, 2005), is well captured, which demonstrates the validity of the detection scheme.

MYR is defined as all extreme rainfall, which is defined as daily rainfall greater than or equal to the local 95th percentile climatological daily rainfall, within 500 km north and south of the position of the MYF after subtraction of tropical cyclone related rainfall (TCR) from the total rainfall. TCR is defined as all rainfall within a 500-km radius of the center of the TC, where the location of the TC is identified by the TRACK algorithm (Hodges et al., 2017). The leftmost column of Fig. 3 shows the climatological monthly mean MYR for different months in the Mei-yu season. The northward propagation of the rain band is well captured in CN05.1.

While many studies in the past have linked LCSMs to MYR on seasonal and monthly time scales (e.g. Wang et al., 2009; Ning et al., 2017; Li et al., 2018), high frequency variability is of critical importance (e.g. Chen et al., 2015; Ding et al., 2020, 2021). Ding et al. (2021) investigated the causes of the record-breaking MYR in 2020 and demonstrated that the WNPSH and other monsoon circulation subsystems experienced several cycles of quasi-biweekly oscillation.

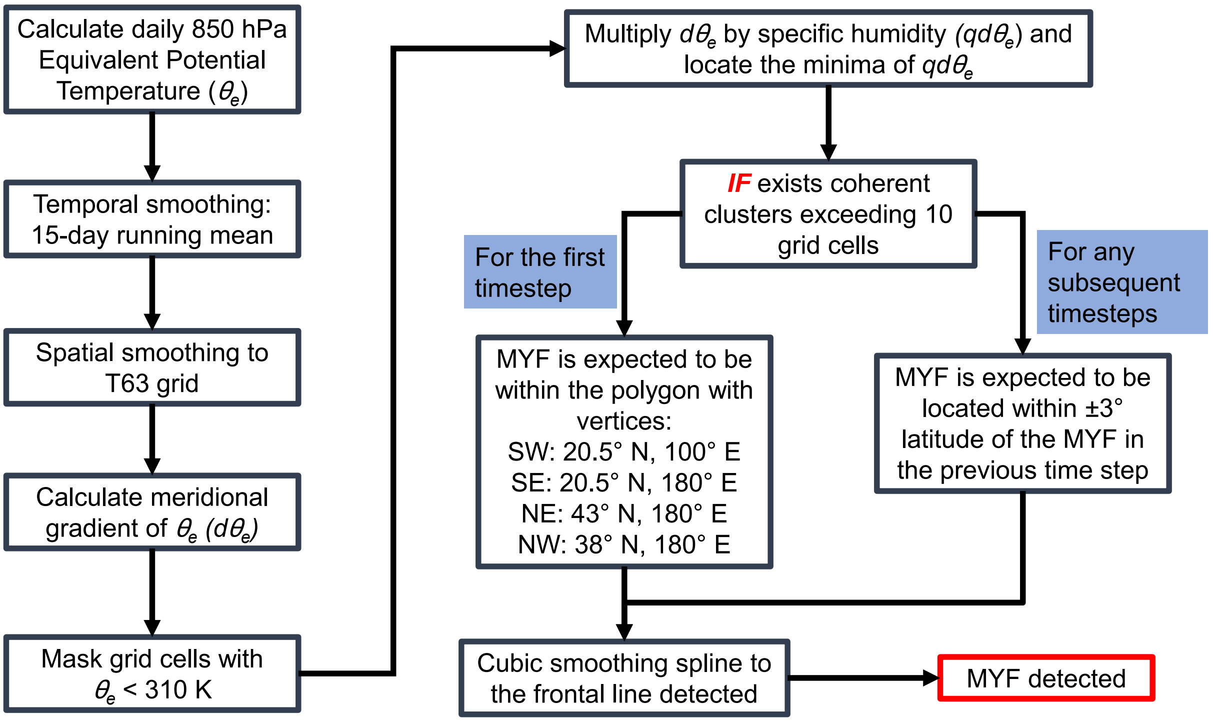

**Fig. 1.** A flowchart for the MYF detection scheme as described in section 3.2 following Befort et al. (2016) and Befort et al. (2017).

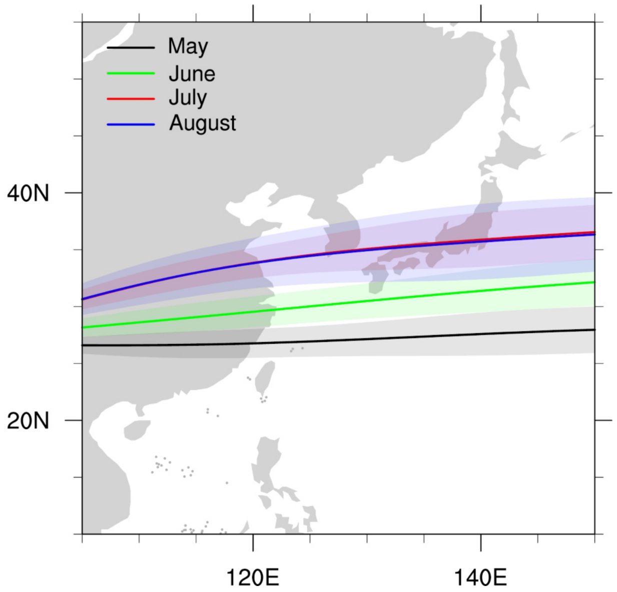

**Fig. 2.** Climatological position of the MYF as identified in ERA5 (1979–2018).

This is one of the reasons that significantly more rainfall was observed in the 2020 Mei-yu season; there were favorable conditions for convective activity development in the YV. To evaluate the impact of the high-frequency variability of LSCMs in a CGSM, two sets of CGSMs have been constructed. CGSM-M is constructed based on monthly indices of LSCM and monthly accumulated MYR for each month in the Mei-yu season (May to August). CGSM-HM is constructed based on half-monthly indices of LSCM and half-monthly accumulated MYR for each half-month in the Mei-yu season.

### 3.3. *Causality-guided statistical model (CGSM)*

The CGSM is constructed using a three-step procedure described as follows: (1) The lagged correlation between detrended anomalies of indices of LSCM and detrended anomalies of MYR for each grid point are calculated. The predictors, which are significantly correlated with MYR at the 0.1 significance level, are considered as potential causal predictors. (2) The modified Peter–Clark (PC) algorithm (Tigramite version 4.2, https://github.com/jakobrunge/tigramite, Runge et al., 2019b; Runge, 2020) is used to evaluate the conditional dependency for all potential causal predictors and MYR. A causal predictor is found if the MYR is shown to be conditionally dependent on the predictor, given other causal predictors. (3) A multiple linear regression model is constructed using all identified causal predictors from step (2) and MYR as the response. The above procedure is repeated for all land grid boxes over continental China for which the number of non-zero MYR entries of the grid box is at least 30. The choice of at least 30 non-zero MYR entries is to avoid construction of the model to predict no MYR.

The significance level of correlation between MYR and predictor pairs in step (1) is chosen to be 0.1, as this is the minimum acceptable significance level. Similar results to those in this study can be obtained if a significance level of 0.05 is used. The significance level of 0.1 is used to maximize the number of potential causally related LSCM–MYR pairs. Causally non-relevant LSCM–MYR pairs would be

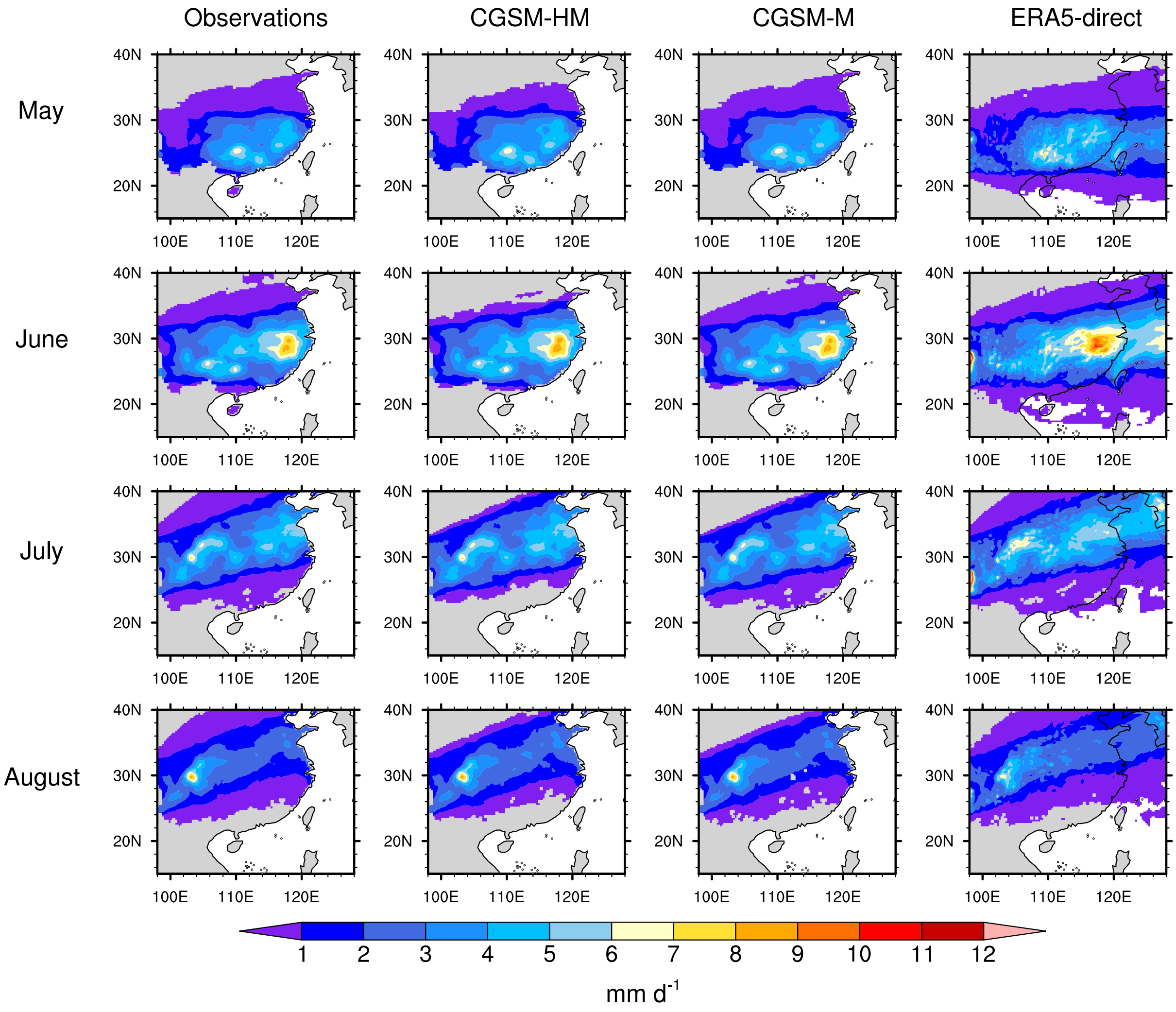

**Fig. 3.** Climatological monthly mean (1979–2018) MYR for May, June, July, and August (top to bottom) from CN05.1 observations, CGSM-HM predictions, CGSM-M predictions, and ERA5 direct output (left to right).

removed by step (2). The conditional dependency in step (2) is obtained, using the modified PC algorithm, by iteratively testing whether the relationship between the potential causal predictor and MYR can be explained by the influence of other causal predictors (see Di Capua et al., 2019 for detailed description). The modified PC algorithm is controlled by two main hyperparameters: (i) maximum time lag with respect to the time of interest and (ii) a regularization parameter, $\alpha_{pc}$ (see Runge et al., 2019b for detailed explanation of the functionality of each hyperparameter). A detailed discussion of the choice of the maximum time lag and $\alpha_{pc}$ can be found later in this section and section 5.1, respectively.

The underlying principle of a CGSM is similar to the studies of Kretschmer et al. (2017) and Di Capua et al. (2019) but with two major differences. (i) The first step of RG-CPD, which was used in Kretschmer et al. (2017) and Di Capua et al. (2019), is to discover new climate indices that have significant association with the response (see Bello et al., 2015 for details). In this study, we are not interested in discovering new indices because we aim to demonstrate the added value and usefulness of the causality approach using known LSCM drivers in explaining the variability of MYR. Consequently, indices of known LSCMs, which are listed in Table 1, are used in this study. (ii) In the studies of Kretschmer et al. (2017) and Di Capua et al. (2019), the responses are area-weighted averages. Consequently, their models are temporal models, which model the regional changes. Due to the importance of the spatial distribution of MYR, such an approach could be a major limitation. In this study, a model is built for each land grid box over continental China. The resultant models can thus capture and explain the spatiotemporal changes of MYR. The total number of CGSM-M and CGSM-HM is 19 188 and 30 059, respectively.

The maximum lag with respect to the time of interest for CGSM-M and CGSM-HM is 11 months and 23 half-months (HMs), respectively. Although the LSCMs chosen in this study could play important roles in part of the underlying physical processes, it is possible that there exist hidden physical processes that are not known yet but are important in controlling MYR. Large maximum time lag aims to assist

**Table 1**. List of the LSCMs considered in the construction of the CGSM.

| LSCM | Definition/References |
|---|---|
| Dipole Mode Index (DMI) | As in Saji et al. (1999); Black et al. (2003) |
| Indian Monsoon Index by Wang and Fan (IMI-WF) | As in Wang and Fan (1999) |
| Indian Monsoon Index by Webster and Yang (IMI-WY) | As in Webster and Yang (1992) |
| ENSO (Niño-3.4) | As in Trenberth (1997) |
| Pacific Japan Pattern (PJ) | As in Nitta (1987); Wakabayashi and Kawamura (2004); Choi et al. (2010); Kim et al. (2012); Li et al. (2014) |
| South Asian High Area Index (SAHI-Area) | As in Ning et al. (2017) |
| South Asian High Northwest Displacement Index (SAHI-NW) | As in Ning et al. (2017) |
| Silk Road pattern principal component 1 (SRP-PC1) | As in Li et al. (2020) |
| Silk Road pattern principal component 2 (SRP-PC2) | As in Li et al. (2020) |
| Sea surface temperature anomaly of Arabian Sea (SSTA-AS) | Mean SST anomaly in the region 10°–25° N, 60°–75° E |
| Sea surface temperature anomaly of Bay of Bengal (SSTA-BoB) | Mean SST anomaly in the region 10°–23° N, 80°–100° E |
| Sea surface temperature anomaly of East China Sea (SSTA-ECS) | Mean SST anomaly in the region 25°–33° N, 120°–130° E |
| Sea surface temperature anomaly of South China Sea (SSTA-SCS) | Mean SST anomaly in the region 10°–23° N, 105°–120° E |
| Western North Pacific Monsoon Index (WNPMI) | As in Wang and Fan (1999); Wang et al. (2001, 2008) |
| Western North Pacific Subtropical High North Index (WNPSH-North) | As in Lu (2002) |
| Western North Pacific Subtropical High West Index (WNPSH-West) | As in Lu (2002) |

the models to capture these hidden processes indirectly. For example, suppose we have a process A(4)→B(2)→C, where the number in the brackets indicates the time lag with respect to C. If we only have the information of A and C, using the causality algorithm with maximum time lag of three, then we would not be able to identify any causal link between A and C. However, if we increase the maximum time lag to five, then we would have captured the causal link between A and C in the absence of B. The use of large maximum lag increases the number of potential predictors significantly, i.e., the number of possible predictors available for CGSM-M and CGSM-HM is 176 and 368, respectively. While this would be an issue in the typical correlation-based variable selection, this is not the case in the causality framework because predictors are only selected if they are causally related to the response as discussed before. Furthermore, if there are several predictors that describe different stages of the same process, only the predictor with the shortest lagged time with respect to the month (or half-month) of interest would be selected by the procedure. The typical number of predictors used to construct CGSM-M and CGSM-HM for all months and half-months is four and seven, respectively, except for the CGSM-HM of the ninth half-month (i.e., first half of May), where typically only six predictors are used. It is also found that predictors related to the PJ, Indian Monsoon Index by Wang and Fan (1999) (IMI-WF), WNPSH North Index (WNPSH-N), WNPMI, and SRP-related indices are frequently selected by CGSM-HM and CGSM-M.

The performances of CGSMs constructed with detrended variables and non-detrended variables are very similar. For the rest of the discussion, we focus on the CGSM constructed with non-detrended variables. Since a CGSM can predict negative values, which have no physical meaning, all predictions of negative values are set to be zero for physical consistency. For model validation, five-fold cross-validation (CV) is used. In five-fold CV, the original data is randomly split into five equal sized subsets. Four subsets are used in model construction, and the remaining subset is used for validation. This procedure is repeated 1000 times with random stratification to achieve statistical robustness.

## 4. Results

Figure 3 shows the spatial distribution of climatological monthly mean MYR (left to right) from observations, modeled using CGSM-HM, modeled using CGSM-M, and from ERA5 direct output over continental China. ERA5 direct output captures the MYR pattern as shown in observations, except MYR in ERA5 direct output appears to be more extreme than that from observations. This is in agreement with Jiao et al. (2021), where it was found that ERA5 overestimated precipitation in summer over China. Both CGSM-HM and CGSM-M show similar climatological patterns to the observed pattern and appear to be better matched with observations than the ERA5 direct output. Although the climatological patterns of CGSM-HM and CGSM-M are very similar (Fig. 3), CGSM-HM has much better performance in capturing variability than CGSM-M (Fig. 4). The median of Pearson's correlation coefficient between observed and model values of CGSM-HM (CGSM-M) for May, June, July, and August, are 0.874 (0.744), 0.847 (0.701), 0.871 (0.754), and 0.858 (0.714), respectively. Figure 4 demonstrates the importance of submonthly variability in understanding MYR, as discussed in the literature (e.g., Ding et al., 2021). Low temporal resolution models (i.e., CGSM-M) would not be able to capture the high frequency variability. In contrast, higher temporal resolution models (i.e., CGSM-HM) have the ability to capture the high frequency variability relevant to MYR related to smaller temporal scale atmospheric phenomena.

Furthermore, the root-mean-square error (RMSE) of CGSM-M is, in general, higher than that of CGSM-HM

(Fig. 5). The largest differences of RMSE between the two models can be found in the middle/lower YV (25°–32.5°N, 110°–120°E) as defined in Martin et al. (2020). This further supports the use of a high temporal resolution CGSM. Sensitivity studies have been performed to investigate the sensitivity of the current method to the definition of extremes from the model construction perspective. The local 80th and 90th percentile thresholds are chosen to be alternative definitions of extremes, as opposed to the 95th percentile (c.f. section 3.2). Similar patterns to those shown in Fig. 4 emerge. This suggests that the current method can construct a model with similar performance for different definitions of extremes. The RMSEs of the five-fold CV for both CGSM-HM and CGSM-M (Fig. 6) show similar patterns to those seen in Fig. 5, which supports the utility of CGSMs.

It is worth pointing out that the region where the largest RMSE (Fig. 5) is found in both CGSM-HM and CGSM-M coincides with the region where the highest climatological monthly mean extreme rainfall is found (Fig. 4). This is because both CGSM-HM and CGSM-M attempt to model accumulated extreme precipitation based on a simple multiple linear regression model, and extreme values tend to be difficult to model by simple multiple linear regression due to their rarity. This can be seen in the results of the five-fold CV (Fig. 6). In the five-fold CV, 32 years of data is used to construct CGSMs, and eight years of data is used to test CGSMs, i.e., less available observations to construct CGSMs. Given that the ability to model extreme MYR is governed by the existence of extreme MYR in the training dataset and extreme MYR is relatively rare, the performances of the CV models are reduced. A possible solution is suggested in section 5.2.2.

To further illustrate the usefulness of CGSMs and the difference between CGSM-M and CGSM-HM, the mean MYR time series for the middle/lower YV has been calculated for observations and for predictions from CGSM-M and CGSM-HM (Fig. 7). In general, the predictions from CGSM-M and CGSM-HM agree with observations relatively well. For the extremes, particularly for 1981, 1995, and 1998, CGSM-HM significantly outperforms CGSM-M. This confirms the importance of capturing high-frequency variability in a CGSM.

## 5. Discussion

### 5.1. *Causality framework*

In theory, the causality approach does not suffer from overfitting because predictors and response are causally related (Kretschmer et al., 2017). In practice, it would depend on the choice of hyperparameter $\alpha_{pc}$ in the causal discovery algorithm, as occasionally slightly different results can be obtained. In this study, we ran the modified PC algorithm (c.f. section 3.3) with five different values of $\alpha_{pc}$, ranging from 0.1 to 0.5, and selected the set of causal predictors that leads to the highest CGSM performance, i.e., the optimal model. This is a useful approach even though it might not be optimal from the causality discovery point of view, as the measure of the goodness of fit is not a measure of causality.

From a physical perspective, LSCMs trigger physical processes that lead to MYR over a large region rather than at a single local grid box. This implies that if CGSMs are constructed based on physical processes rather than purely statistical associations, the predictors in CGSMs should have a certain degree of spatial consistency, i.e., the locations of the grid boxes that select certain predictors should form coherent large clusters. As demonstrated in Fig. 8, the LSCMs that are used in CGSMs (e.g., IMI-WF with five half-month lag) form large coherent spatial clusters. Thus, these LSCMs are linked to the regional MYR through physical processes, and, consequently, CGSMs are not purely statistical models but they have physical significance.

Under the causality framework, the predictors in CGSMs correspond to different processes. Some of these predictors could be linked to the following hypotheses: (1) Liu and Ding (2008) investigated the teleconnection between ISM precipitation and Mei-yu rainfall over YV. They reported the southwest–northeastward teleconnection mode originated from southwestern India, and it propagates through the Bay of Bengal to the YV and southern Japan. The IMI-WF predictor (lag five) could correspond to the preconditions of ISM onset. (2) Ning et al. (2017) hypothesized about the influence of the dynamical changes of the south Asian high (SAH) on MYR. When the SAH expands and intensifies, the WNPSH also intensifies and extends westward. More moisture is transported to the YV region. That combined with other environmental factors leads to more extreme rainfall in the region. The SAHI-Area predictor (lag two) potentially reflects this mechanism. However, identifying the actual physical processes that relate predictors and response is beyond the scope of the current study.

### 5.2. *Limitations*

There are three noticeable limitations in the current approach where potential improvement in the future could be possible.

#### 5.2.1. *Incomplete set of LSCMs*

While a significant number of LSCMs are considered in the CGSM construction, not all potentially relevant LSCMs, such as LSCMs that control long-term, low-frequency variability, are included. Ding et al. (2008) found that the regional rainfall in the EASM region exhibits multidecadal variability with the most dominate mode being ~80 years. Other higher-frequency multidecadal oscillations are also important in controlling regional variability. Furthermore, Ding et al. (2008) has shown that there is a shift in the meridional precipitation pattern from the tripole pattern in 1951–78 to the dipole pattern in 1993–2004. This is also related to variations in the large-scale circulation and the thermodynamic and moisture fields in the EASM region in the same period. Given that the predictor set of the CGSM does not include any multidecadal LSCMs, it could be problematic

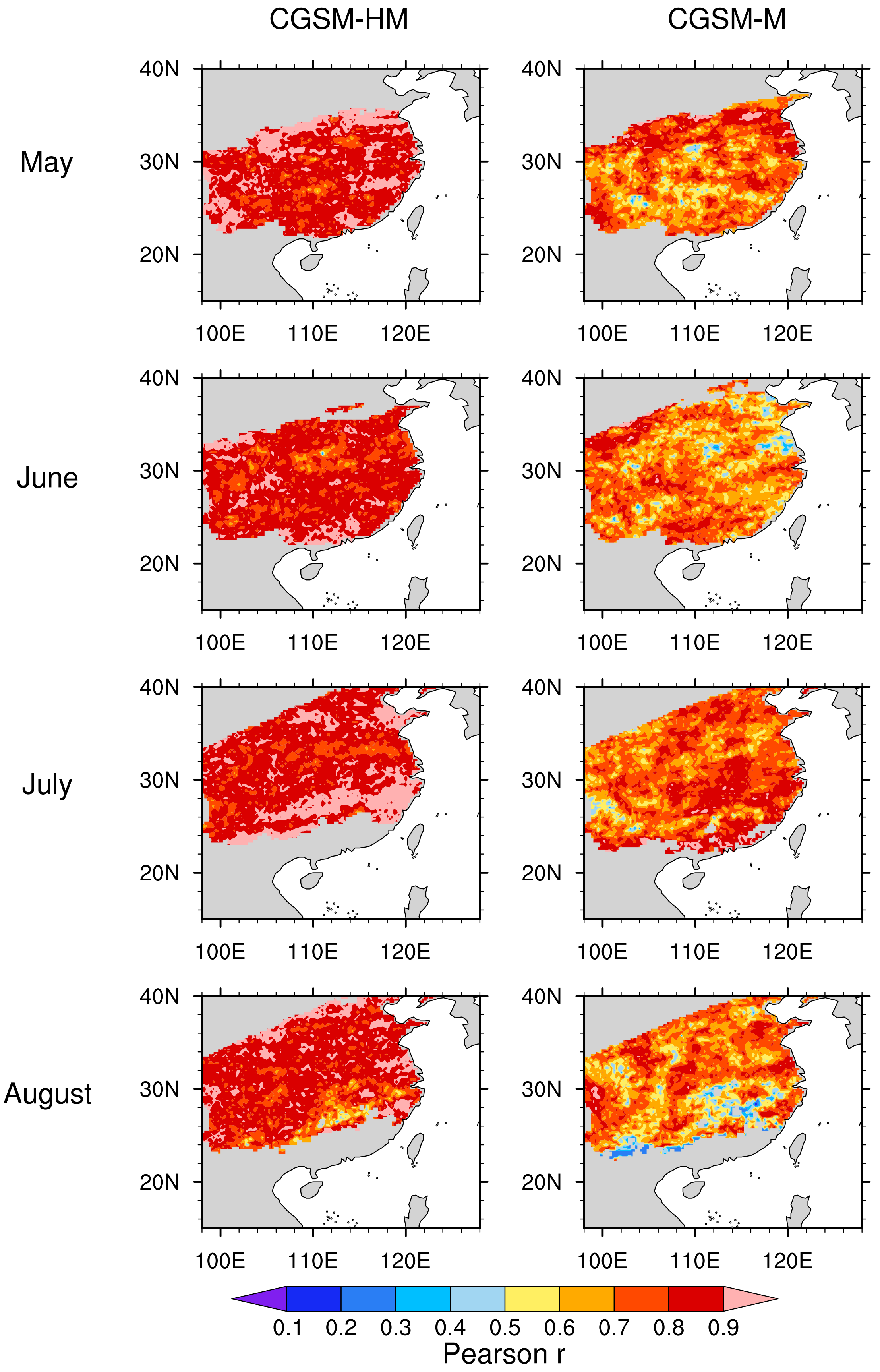

**Fig. 4.** Map of Pearson correlation coefficient between CN05.1 and model values of monthly accumulated MYR calculated using CGSM-HM (left column) and CGSM-M (right column) for May, June, July, and August (from top to bottom) for the period 1979–2018.

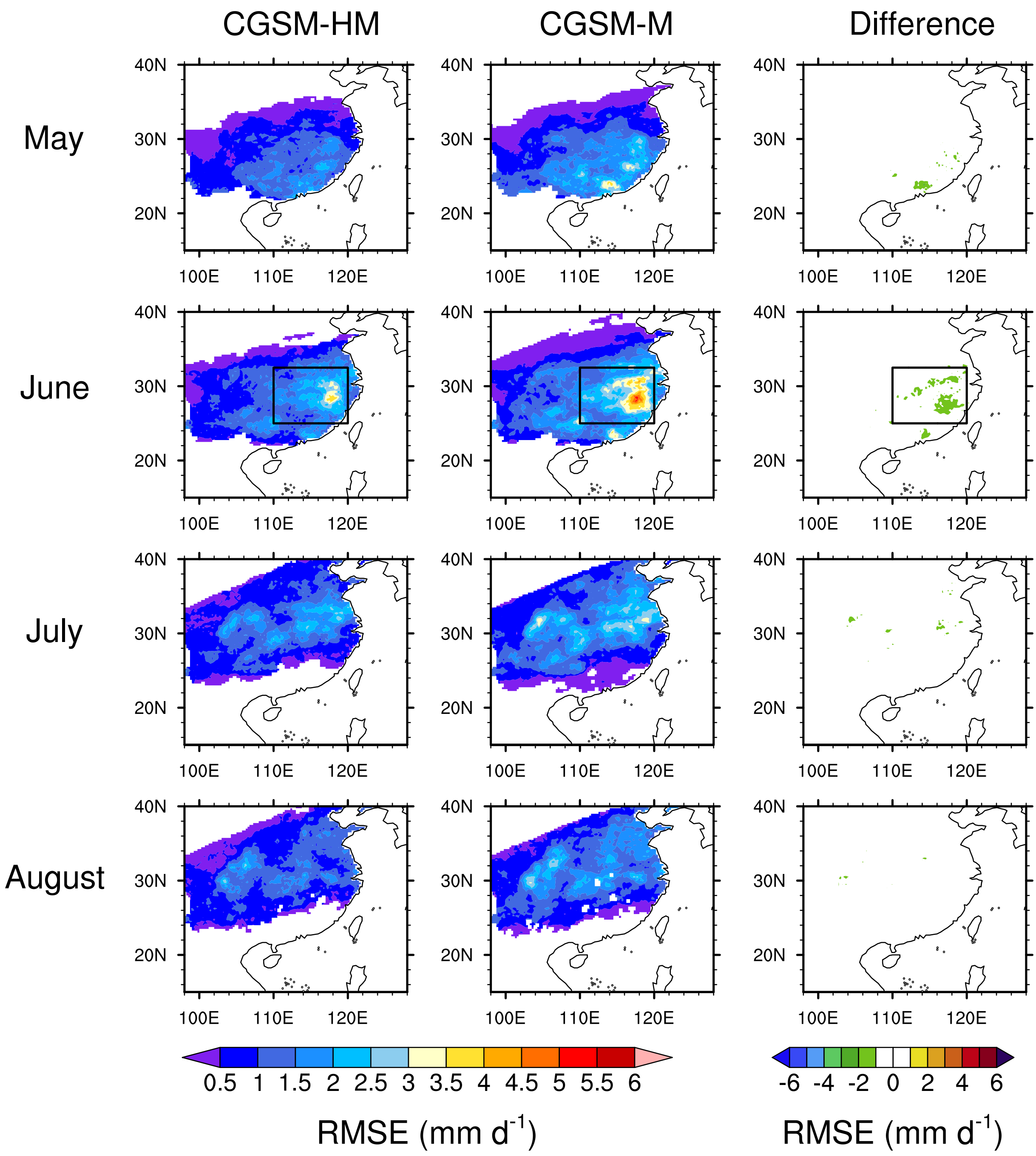

**Fig. 5.** RMSE of the optimal models for CGSM-HM (left), CGSM-M (middle), and the difference between CGSM-HM and CGSM-M (right) for the period 1979–2018. The black box indicates the middle/lower Yangtze River region as defined by Martin et al. (2020).

to apply the current CGSMs to a different climatic state. To evaluate the performance of the CGSM in a different climate state, similar analyses have been done using data from ERA5 BE and CN05.1 in the period of 1961–78. A consistency check based on the climatological monthly MYR pattern between ERA5 BE and CN05.1 has been done. While the general patterns appear to be similar, the region of high-intensity MYR in ERA5 BE is much larger and extreme than that in CN05.1 (not shown). Possible explanations could be changes in the global observation system in 1979, as was found in ERA-40 (Bengtsson et al., 2004), and the bias in simulated precipitation in the ECMWF IFS (Lavers et al., 2021).

Over the study region, the overwhelming advantage of the high-temporal-frequency-resolution model has been diminished (Fig. 9). Although there are patches of regions with relatively high positive Pearson's correlation coefficient, the median of Pearson's correlation coefficient between observed values and model values (Fig. 9) of both CGSM-HM and CGSM-M is close to zero for the Mei-yu season.

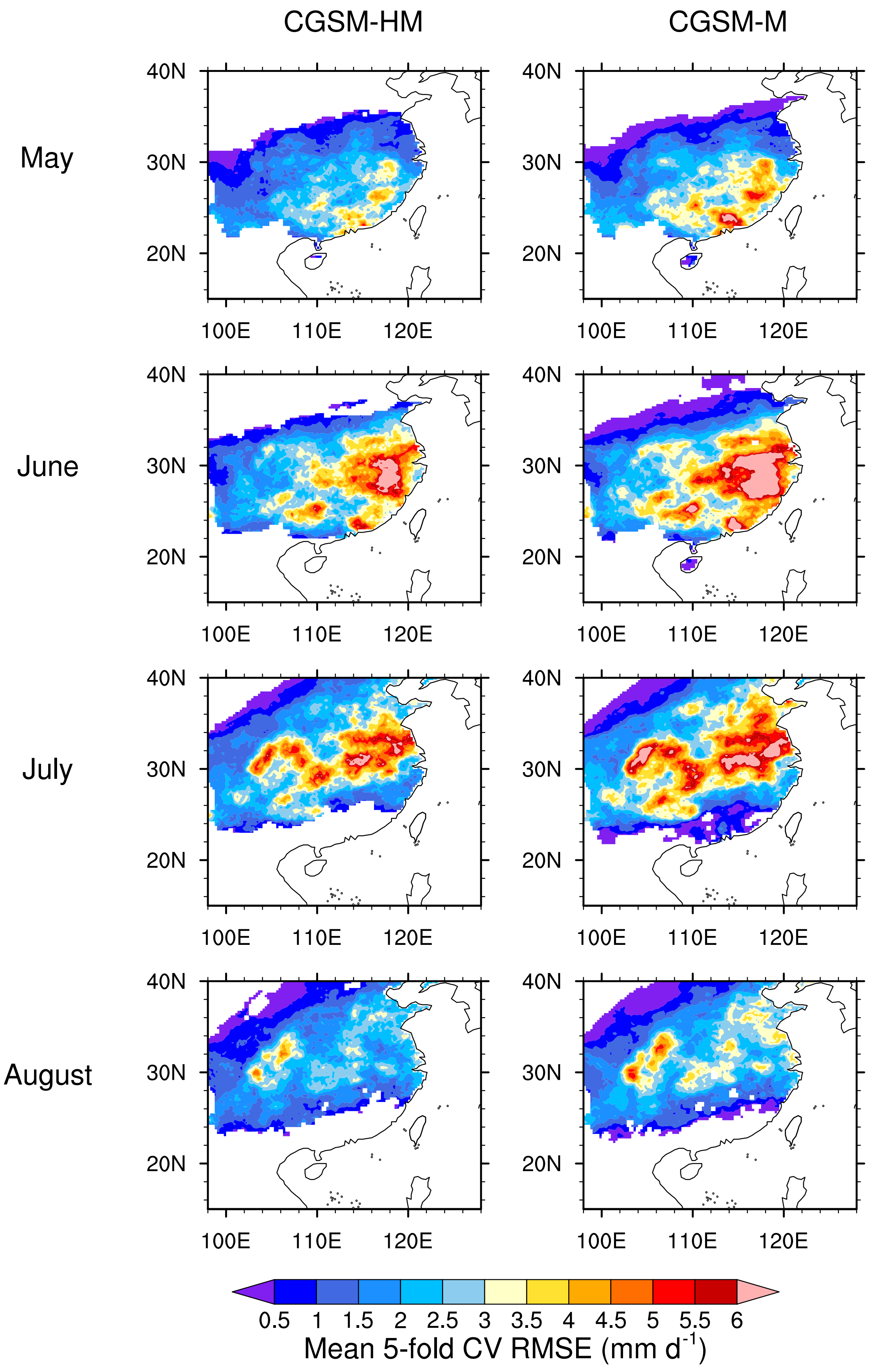

**Fig. 6.** Mean RMSE of five-fold cross-validation of the CGSM-HM (left column) and CGSM-M (right column) for the period 1979–2018.

This shows that the CGSM, which is trained with data from the period 1979–2018, does not have the ability to capture variability in the entire study region for the period 1961–78. The CGSM-HM has a smaller RMSE than the CGSM-M in July within the middle/lower YV region (Fig. 10). The location of high RMSE in CGSM-HM and CGSM-M appears to be similar (e.g., Guangdong province in May and June, and along the lower/middle YV in July). This could indicate that some of the key physical processes are not captured by the current statistical models. This might be related to the limited number of LSCMs considered in this study as well as the potential differences in processes in the period 1961–78. Furthermore, in general, the mean five-fold CV RMSE of CGSMs using data from 1979–2018 (Fig. 6) is lower than the RMSE of CGSMs using data from 1961–78 (Fig. 10), except for in the lower/middle YV in June. This shows the possible shift between the spatial distribution of MYR in the period 1961–78 and that in the period 1979–2018. Given that the predictor set of the CGSM does not include any LSCM that controls multidecadal variability, it would not be surprising that the CGSM does not have the best performance in predicting the MYR in the period 1961–78.

### 5.2.2. *Limited available observations*

The CGSMs constructed in this study aim to model MYR over continental China. Currently the models are built based on 40 years of observational and reanalysis data. As briefly discussed in section 4, the performance of the model is limited by the amount of available extreme observations, as demonstrated in Fig. 6. This can be improved if more data are available. However, as shown in section 5.1, using a longer historical record might not improve the model due to the influence of long-term variability and climatic shifts, and possible observational biases in the presatellite era. One potential approach to address this issue is to make use of the Osinski–Thompson approach (Osinski et al., 2016; Thompson et al., 2017) with an application of a bias correction technique. The Osinski–Thompson approach has been shown to be extremely useful in studies of extreme windstorm events (Osinski et al., 2016; Walz and Leckebusch, 2019; Angus and Leckebusch, 2020; Ng and Leckebusch, 2021) and extreme rainfall events (Thompson et al., 2017). The underlying principle is to make use of different atmospheric states, which are produced by state-of-the-art general circulation models (GCMs), in a well-defined climate state. Since these GCMs are governed by physical equations, the physical causal relationship between LSCMs and MYR should be consistent with the observations. This way, we could increase the number of extreme "observations" by at least 100-fold. Although the rainfall simulated in GCMs is typically biased relative to observations because it is affected by parameterization schemes as well as other numerical issues, a bias correction technique can be used to address this issue. Consequently, CGSMs, which are developed using the underlying relationships between LSCMs and MYR and the Osinski–Thompson approach, may be more robust.

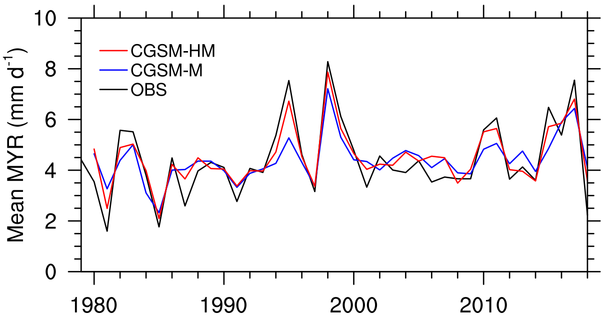

**Fig. 7.** Time series of mean MYR from observations (black line), prediction by CGSM-M (blue line), and prediction by CGSM-HM (red line) in the middle/lower YV (black box in Fig. 5) for June from 1979–2018.

### 5.2.3. *Optimization of predictor selection*

Although the predictors selected in the causality framework should be physically linked to the response, the set of selected predictors could be sensitive to the choice of hyperparameter in the causality algorithm, as discussed before. One approach to improve the stability and reliability of the causality-based predictor selection procedure is to make use of the fact that selected predictors have to be spatially consistent (Fig. 8). For example, if a predictor is selected for a particular grid box but not for the neighboring grid box, then this predictor is said to be spatially inconsistent and would be removed from the final model. On the other hand, if a predictor is selected for a particular grid box and the grid box is part of a large, spatially coherent cluster, then this predictor would be kept in the final model. This approach can improve the

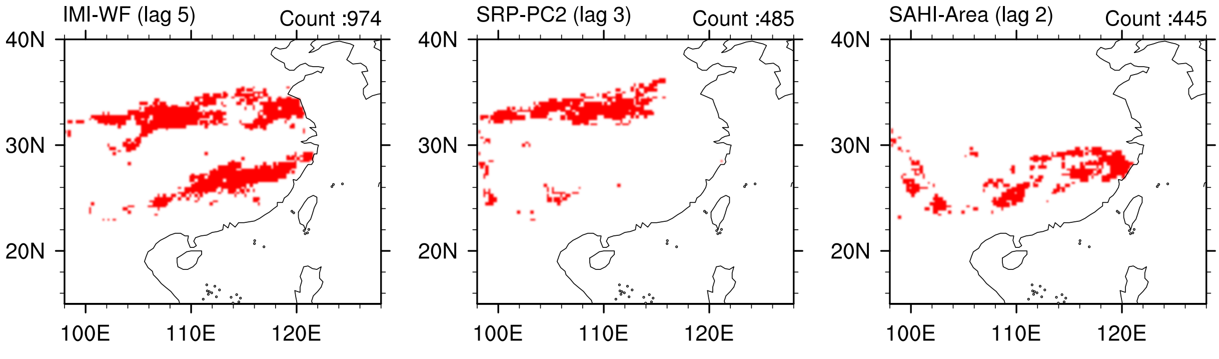

**Fig. 8.** Map of the three most frequently chosen predictors for the second half of June.

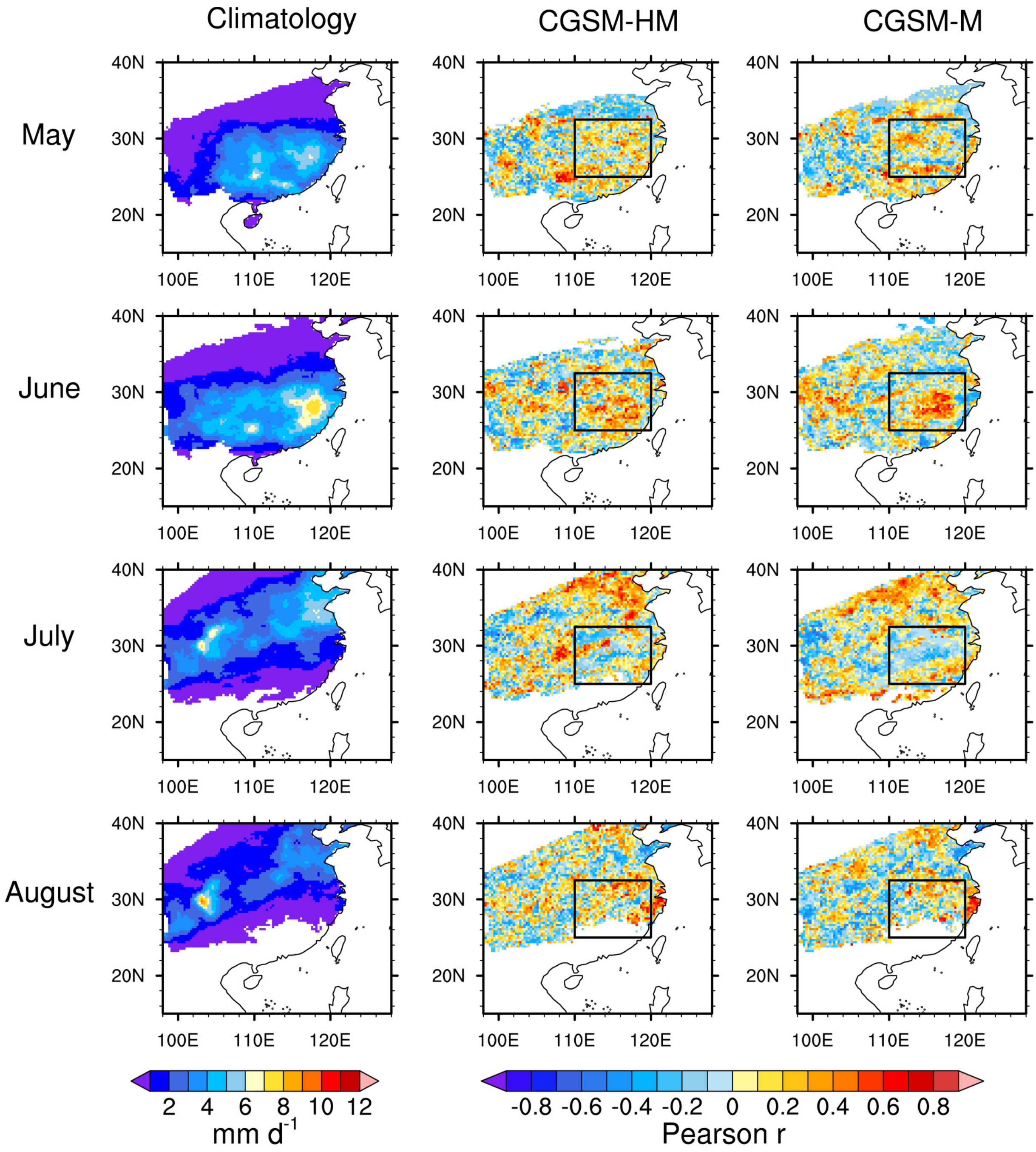

**Fig. 9.** (Left) Climatological MYR for May, June, July, and August in the period 1961–78. Map of Pearson correlation coefficient between observations and predictions from CGSM-HM (middle) and CGSM-M (right) for May, June, July, and August (top to bottom) using data in the period of 1961–78. The black box indicates the middle/lower YV as defined by Martin et al. (2020).

physical consistency in predictor selection procedure.

## 6. Conclusions

This is a pilot study for the potential application of a data-driven, causality-guided statistical approach to explore the relationship between extreme Mei-yu rainfall (MYR) and known large-scale climate modes (LSCMs) through the construction of a prediction model based on the causal relationships. Using the causality approach, we have demonstrated that known LSCMs from literature can be used to model MYR with good accuracy (Fig. 4). Since the causality approach does not lead to overfitting due to the inclusion of noncausal-related predictors in the model, as shown in Kretschmer et al. (2017), the approach is reliable in identifying important LSCMs, which causally control MYR. Furthermore, the causality-guided statistical approach shows the importance of capturing high-frequency variability by using

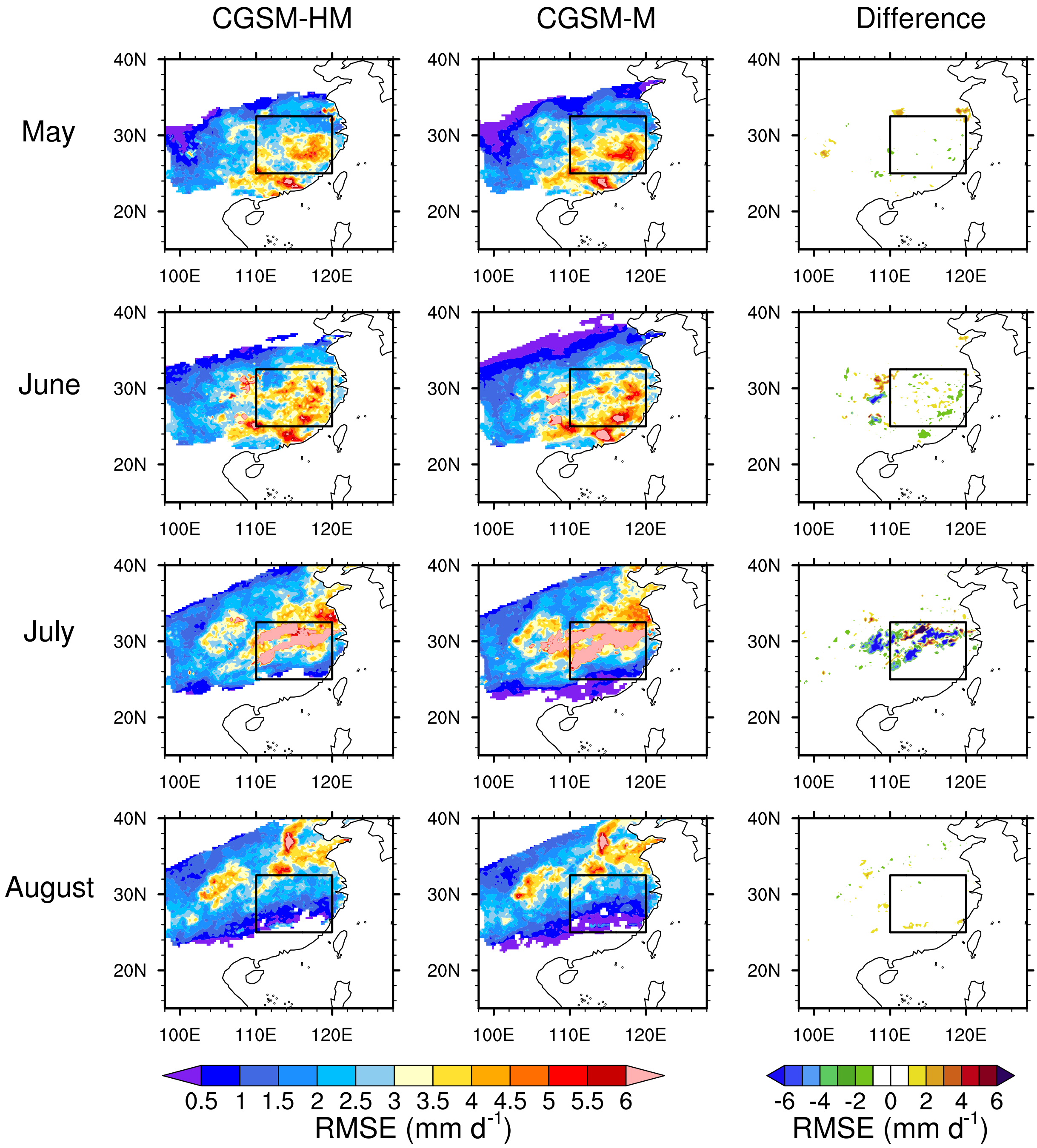

**Fig. 10.** RMSE of the optimal models for CGSM-HM (left), CGSM-M (middle), and the difference between CGSM-HM and CGSM-M (right) for May, June, July, and August (top to bottom) using data in the period of 1961–78. The black box indicates the middle/lower YV as defined by Martin et al. (2020).

high-temporal-resolution predictors and responses to model the MYR, which is in good agreement with the literature (e.g., Chen et al., 2015; Ding et al., 2020, 2021). Based on the causality approach, it is possible to identify the main LSCMs controlling MYR in specific regions based on the spatially consistent pattern of selected predictors (Fig. 8). Known limitations of this approach have also been discussed in detail, and methods to address these limitations have been suggested (section 5.2).

While we have demonstrated the causality-guided approach is useful in constructing models by uncovering the underlying causal physical relationship between LSCMs and MYR, a detailed investigation of the underlying physical processes that are driven by LSCMs to influence MYR is beyond the scope of this study and should be explored further once a full causally explanatory model is identified. For this, further investigations on the underlying physical processes can be done using the causality approach by combining the method described in this study and response-guided community detection (Bello et al., 2015), and, consequently, the dynamical relationship between LSCMs and MYR in different stages of the Mei-yu season in a given region could be revealed. This can then optimize the index, which represents a LSCM, and at the same time provide necessary physical understanding of the index.

This study shows the importance of LSCMs in modelling MYR. This is consistent with the analysis, which was done by Martin et al. (2020), on the source of skill in predicting June mean EASM rainfall over China using GloSea5. They identified that the main source of skill comes from the equatorial SST from the preceding winter by influencing the WNPSH. Using the causality approach, the importance of LSCMs at different stages of the EASM can be identified in a more comprehensive and efficient manner. The statistical model constructed based on predictors, which are selected using the causality approach, have high explanatory power as well as transferability. This study also indicates that the predictability of the seasonal forecast of EASM rainfall can be improved by improving the forecast ability of relevant LSCMs. Ultimately, the causality approach can play an important role in improving the prediction of MYR in the future climate due to its ability to uncover the intimate relationship between LSCMs and MYR.

***Acknowledgements***. The authors thank two anonymous reviewers and an associate editor-in-chief for their valuable comments. This work was supported by the UK-China Research and Innovation Partnership Fund through the Met Office Climate Science for Service Partnership (CSSP) China as part of the Newton Fund. The authors thank Dr. Jia WU at National Climate Center, China Meteorological Administration for providing CN05.1. The calculations described in this paper were performed using the BlueBEAR HPC service at the University of Birmingham.

***Electronic supplementary material:*** Supplementary material is available in the online version of this article at https://doi.org/10.1007/s00376-022-1348-3.

***Open Access*** This article is licensed under a Creative Commons Attribution 4.0 International License, which permits use, sharing, adaptation, distribution and reproduction in any medium or format, as long as you give appropriate credit to the original author(s) and the source, provide a link to the Creative Commons licence, and indicate if changes were made. The images or other third party material in this article are included in the article's Creative Commons licence, unless indicated otherwise in a credit line to the material. If material is not included in the article's Creative Commons licence and your intended use is not permitted by statutory regulation or exceeds the permitted use, you will need to obtain permission directly from the copyright holder. To view a copy of this licence, visit http://creativecommons.org/licenses/by/4.0/.

## Electronic Supplementary Material to

A Causality-guided Statistical Approach for Modeling Extreme Mei-yu Rainfall Based on Known Large-scale Modes—A Pilot Study
