# Supplementary material for: A Causality-guided Statistical Approach for Modeling Extreme Mei-yu Rainfall Based on Known Large-scale Modes—A Pilot Study
Source: Adv Atmos Sci. 2022 May 14;39(11):1925–40. doi: 10.1007/s00376-022-1348-3 (PMC9107216; doi:10.1007/s00376-022-1348-3)
Supplement: Supplementary file 1 — A Causality-guided Statistical Approach for Modeling Extreme Mei-yu Rainfall Based on Known Large-scale Modes—A Pilot Study [file 376_2022_1348_MOESM1_ESM.pdf]

Electronic Supplementary Material to:
A Causality-guided Statistical Approach for Modeling Extreme Mei-yu Rainfall Based on Known Large-scale Modes—A Pilot Study*

Kelvin S. NG[1], Gregor C. LECKEBUSCH[1], and Kevin I. HODGES[2]

[1]*School of Geography, Earth and Environmental Sciences, University of Birmingham, Birmingham B*15 2*TT, United Kingdom*

[2]*Department of Meteorology and NCAS, University of Reading, Reading RG*6 6*BB, United Kingdom*

ESM to: Ng, K. S., G. C. Leckebusch, and K. I. Hodges, 2022: A causality-guided statistical approach for modeling extreme Mei-yu rainfall based on known large-scale modes—A pilot study. *Adv. Atmos. Sci.*, **39**(11), 1925−1940, https://doi.org/10.1007/s00376-022-1348-3.

*The online version of this article can be found at https://doi.org/10.1007/s00376-022-1348-3.

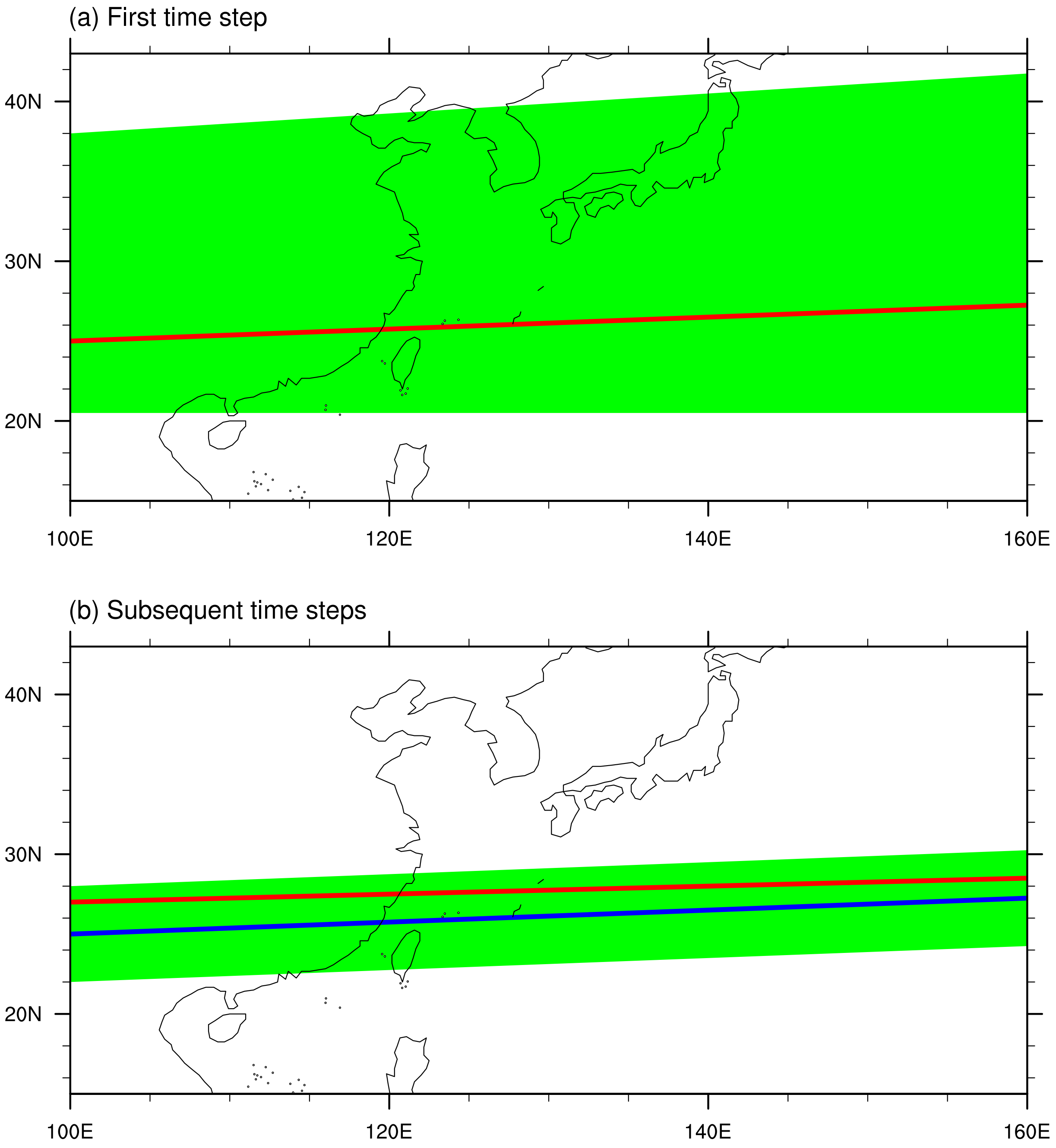

Fig. S1. Schematic diagram of MYF detection (a) for the first time step and (b) for the subsequent time steps (see section 3.2 and Fig. 1 for more information). Green regions indicate the search regions. Red lines indicate the MYF detected at the current time step. Blue line indicates the MYF detected at the previous time step.
